# Interrater Agreement between Bedside and Video Raters Using the CPOT-Neuro for Pain Assessment in Critically Ill Patients with a Brain Injury

**DOI:** 10.3390/nursrep14010017

**Published:** 2024-01-19

**Authors:** Vivienne Nguyen, Melissa Richard-Lalonde, Céline Gélinas

**Affiliations:** 1 Undergraduate Neuroscience Program, McGill University, Montreal, QC H3A 1R1, Canada; vien-an.nguyen@mail.mcgill.ca; 2 Ingram School of Nursing, McGill University, Montreal, QC H3A 2M7, Canada; melissa.richard-lalonde@mail.mcgill.ca; 3 Centre for Nursing Research and Lady Davis Institute, Jewish General Hospital—CIUSSS West-Central-Montreal, Montreal, QC H3T 1E2, Canada

**Keywords:** interrater agreement, pain behavior assessment, video rating, critically ill, brain injury

## Abstract

This study aimed to examine the interrater agreement of Critical-Care Pain Observation Tool-Neuro (CPOT-Neuro) scores as a newly developed tool for pain assessment in patients with critical illness and brain injury between raters using two methods of rating (bedside versus video) during standard care procedures (i.e., non-invasive blood pressure and turning). The bedside raters were research staff, and the two video raters had different backgrounds (health and non-health disciplines). Raters received standardized 45 min training by the principal investigator. Video recordings of 56 patient participants with a brain injury at different levels of consciousness were included. Interrater agreement was supported with an Intraclass Correlation Coefficient (ICC) > 0.65 for all pairs of raters and for each procedure. Interrater agreement was highest during turning in the conscious group, with ICCs ranging from 0.79 to 0.90. The use of video recordings was challenging for the observation of some behaviors (i.e., tearing, face flushing), which were influenced by factors such as lighting and the angle of the camera. Ventilator alarms were also challenging to distinguish from other sources for the video rater from a non-health discipline. Following standardized training, video technology was useful in achieving an acceptable interrater agreement of CPOT-Neuro scores between bedside and video raters for research purposes.

## 1. Introduction

A pain assessment in patients with a critical illness and a brain injury is challenging, as many of them may present with an altered level of consciousness (LOC). Although brain tissue itself is not sensitive, pain can arise from lesions on the external structures of the brain and other sources, such as invasive equipment necessary for mechanical ventilation [1] and standard care procedures common in the intensive care unit (ICU) [2]. Some standard care procedures may act as noxious stimuli, leading to nociception and pain. Nociception is the neural physiologic process of encoding noxious stimuli that may trigger autonomic (e.g., increased heart rate) or behavioral (e.g., motor withdrawal reflex) responses [3]. Nociception may also lead to pain, which is a personal experience that is best described by the person experiencing it. The patient’s self-report is considered as the “reference standard” measure for pain, which should be accepted and respected [3]. However, in the ICU, patients with a brain injury often cannot express their pain through verbal self-reporting due to altered LOC, sedation, or mechanical ventilation that may alter their ability to communicate [4]. This inability to self-report does not negate the possibility that a patient experiences pain [3]. In such a situation, nonverbal behaviors provide vital information [3], and behavioral assessment tools are recommended as an alternative reference method for pain assessment [4,5].

Several behavioral assessment tools currently exist for critically ill adults [6]. The Behavioral Pain Scale (BPS) [7] and the Critical-Care Pain Observation Tool (CPOT) [8] have been widely validated among ICU patients with various diagnoses; however, the content of these tools may be less applicable in patients with a brain injury and with an altered LOC [4]. Indeed, patients with a critical illness and brain injury exhibit various behaviors indicative of pain that may differ from behaviors exhibited by medical or surgical ICU patients. For instance, the contraction of the upper face (i.e., brow lowering, orbit tightening) was more frequently observed than the contraction of the lower face (i.e., upper lip raising, clenched teeth) or a simultaneous contraction of the upper and lower face (grimace) in ICU patients with a brain injury [9,10]. Also, tearing and face flushing were newly described in ICU patients with a brain injury [9,10]. Therefore, refinements to these tools are indicated for a more accurate detection of pain in this vulnerable patient group.

Based on this evidence, the content of the CPOT was adapted to refine the description of behaviors and their scoring and to include other behaviors indicative of pain in patients with a critical illness and a brain injury. The refined scale is called the CPOT-Neuro, which was recently validated in a multi-site study [11]. Our findings supported the ability of the CPOT-Neuro to discriminate between standard care procedures, which could be related to noxious (e.g., turning, endotracheal suctioning) or non-noxious stimuli (e.g., soft touch, non-invasive blood pressure [NIBP]) [6] as well as correlating with self-reported pain intensity. The interrater agreement between two bedside raters (i.e., research staff and ICU nursing staff) was also supported [11]. As part of this research study, patient participants were video-recorded during standard care procedures. In this report, we aimed to examine the interrater agreement of CPOT-Neuro scores between raters using two methods of rating (bedside versus video) in the context of an undergraduate student project. Guidelines for reporting reliability and agreement studies (GRRAS) were followed [12]. 

## 2. Materials and Methods

### 2.1. Design

A prospective cohort with repeated-measures design was used for the larger validation study [11]. This design allowed the collection of data across non-noxious and noxious procedures as part of standard care. To achieve the study objective, NIBP and turning were selected as non-noxious and noxious procedures, respectively, as these are commonly performed in ICU patients with a brain injury. Both procedures were performed as part of standard care on the same day by ICU nursing staff. Video recordings with a view of the patient’s face and body were included in this interrater agreement study. 

### 2.2. Sample and Setting

A sample size greater than 33 patients was required to detect a minimal Intraclass Correlation Coefficient (ICC) value of 0.60 between pairs of two raters with an alpha of 0.05 and a power of 80% [13]. Video recordings of patients were performed in two university ICU settings from large tertiary trauma centers in Montreal with similar bed capacities, admitting, on average, 1000–1500 patients annually. We used a subsample of the larger validation study [11]. Using consecutive sampling, patients were considered eligible if they (1) were 18 years or older; (2) were admitted for brain injury (trauma-related or not) to the ICU for less than 4 weeks; (3) and had a Glasgow Coma Scale (GCS) score ≥ 4. Patients were excluded if they had sustained a spinal cord injury affecting the motor activity of the four limbs, had cognitive deficits or psychiatric conditions prior to the brain injury, were previously diagnosed with epilepsy, were receiving neuromuscular blocking agents, had a score of −5 (unarousable) on the Richmond Agitation Sedation Scale (RASS), or had suspected brain death. This study was approved by the research ethics committee of each participating site (#15-994 at site 1 and #2015-1164 at site 2). Patients capable of consenting or representatives qualified to consent for suddenly incapable patients provided written consent for this research study, and incapable patients who later recovered their ability to consent provided informed written consent.

### 2.3. Procedures

Video recordings were captured for the duration of procedures at the bedside using two cameras: (a) one for the face view (held by the research staff) and (b) one for the body view (on a tripod at the foot of the bed). As described in the larger study [11], patient participants were assessed for pain using the CPOT-Neuro by the research staff at the bedside. To achieve this study objective, patient participants were also assessed for pain with the CPOT-Neuro using their video recordings which were viewed independently by two video raters (VR): (a) one undergraduate student trainee in neuroscience newly trained in 2022 (VR1—VN) and (b) one trained research staff (PhD candidate in nursing) with 7 years of experience (VR2—MRL) using the CPOT and the CPOT-Neuro [11,14]. Interrater agreement between the CPOT-Neuro scores of the two video raters and the bedside raters (research staff member) during NIBP and turning was examined. The bedside raters involved 5 research staff members, including an experienced clinical research coordinator, 3 undergraduate nursing trainees, and a graduate nursing trainee (who was also involved as VR2). 

### 2.4. Instruments

#### 2.4.1. Critical-Care Pain Observation Tool–Neuro

**Figure 1:**
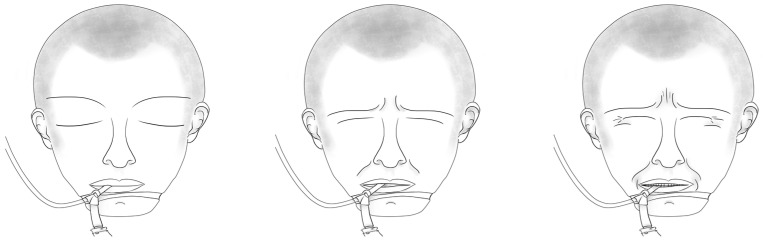

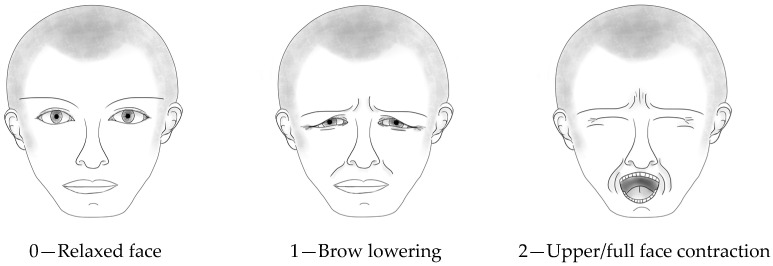
Facial expression scoring in CPOT-Neuro. © Céline Gélinas.

We used the CPOT-Neuro to assess pain in patient videos during the non-noxious and noxious procedures. The CPOT-Neuro development was previously described [11]. Briefly, this tool includes the following five behavioral items: (a) facial expression, (b) body movements, (c) ventilator compliance or vocalization (based on the patient’s condition, i.e., mechanically ventilated or not), (d) muscle tension, and (e) autonomic responses (tearing and/or face flushing). Facial expression (inspired by key facial responses to pain [15,16]), body movements, and ventilator compliance/vocalization are rated from 0 to 2 (see Figure 1 for facial expression), while muscle tension and autonomic responses are rated as absent (0) or present (1). Muscle tension is evaluated by performing a passive flexion and extension of the patient’s arm to feel any resistance to movements. The total score can range from 0 to 8, similar to the scoring of the original CPOT [8]. The validity of its use in ICU patients with a brain injury was supported by higher CPOT-Neuro scores during noxious compared to non-noxious procedures and a moderate positive correlation (Spearman rho = 0.63) with self-reported pain intensity during turning [11]. The interrater agreement of the CPOT-Neuro scores obtained during bedside turning between research staff and ICU nursing staff was supported with an ICC of 0.76 (95% confidence interval (CI): 0.68–0.82) [11].

Bedside raters and the two video raters (VR1 and VR2) were trained by the principal investigator and author of the CPOT-Neuro (CG). This 45 min training was previously described [14]. Briefly, the training included the description of the tool’s items and scoring, as well as patient videos to practice scoring with the CPOT-Neuro and a competence test with three selected patient videos. Exact CPOT-Neuro scores or a difference of no more than one point were acceptable to pass the competence test.

#### 2.4.2. Sociodemographic and Clinical Variables

Sociodemographic data were collected from each participant, including age, sex, and ethnicity. Clinical data were collected from the patient’s medical chart, including diagnosis and the severity of illness (Acute Physiology And Chronic Health Evaluation II [APACHE II]) [17], and level of consciousness (GCS score). The GCS was used to evaluate the patient’s LOC, which was clustered into 3 main categories: (a) unconsciousness (GCS ≤ 8), (b) altered LOC (GSC = 9–12), and (c) consciousness (GCS = 13–15) [18].

#### 2.4.3. Data Analysis

SPSS software (version 24.0) was used for data analysis. Descriptive statistics (i.e., frequency, median, and interquartile range (IQR)) were calculated for CPOT-Neuro scores and sociodemographic and clinical data. The ICC (two-way mixed model) was used to examine the interrater agreement between the CPOT-Neuro scores of the bedside and video raters. A minimal ICC > 0.60 was required to represent the acceptable interrater agreement for research purposes [12]. 

## 3. Results

### 3.1. Sample Description

In total, videos of 56 patient participants (31 conscious, 17 altered LOC, 8 unconscious) were assessed using CPOT-Neuro. Participants were middle-aged, and the majority were Caucasian males admitted to the ICU for a traumatic brain injury (TBI). Patients with an altered LOC or who were unconscious were unable to self-report their pain (45% of the sample), and most of them were mechanically ventilated. APACHE II mean scores tended to increase with lower levels of consciousness, indicating the severity of injury. The patient sample is described in Table 1. 

### 3.2. CPOT-Neuro Scores of the Bedside and Video Raters

Low CPOT-Neuro scores were found during NIBP, and high scores were found during turning. Based on the descriptive statistics of CPOT-Neuro scores (Table 2), there seemed to be some differences between raters, especially during turning. VR1 provided the lowest CPOT-Neuro scores during turning in unconscious patients. 

### 3.3. Interrater Agreement of CPOT-Neuro Scores between Pairs of Bedside and Video Raters 

The interrater agreement coefficients of CPOT-Neuro scores between pairs of bedside and video raters for each procedure are described in Table 3. ICCs for the pairs of bedside and VR2 raters were calculated using a smaller sample (*n* = 35), considering that VR2 was also involved as the bedside rater for 21 patient participants. The ICCs for all three pairs of raters were > 0.65 for the total sample (*n* = 56). ICCs were also acceptable in each of the LOC groups except for the pair of the bedside and VR1 raters during NIBP in the altered LOC group with a low ICC of 0.44. Of note, ICC could not be obtained for the pair of bedside VR2 raters in the unconscious group due to the very small sample (*n* = 3). The interrater agreement was highest for all pairs of raters during turning in the conscious group, with ICCs ranging from 0.79 to 0.90. 

## 4. Discussion

This is the first study reporting on the interrater agreement of CPOT-Neuro using video recordings. Overall, the interrater agreement between pairs of bedside and video raters was supported with ICCs > 0.60, which is appropriate for research purposes [12] and considering that different methods were used (bedside rating versus video rating). Overall, ICCs were higher during turning (0.74–0.80) compared to NIBP (0.65–0.69). A similar ICC (0.76) was also reported during turning in the larger validation study between bedside raters (research staff and ICU nursing staff) [11]. Interestingly, ICCs tended to be higher in the conscious group compared to the altered LOC and unconscious groups. Conscious ICU patients with a brain injury are more likely to exhibit common pain behaviors such as a grimace, whereas those who are unconscious or with an altered LOC express a variety of subtle behaviors that may be indicative of pain [1,9,10]. 

The use of video technology in behavioral observation is a key method for collecting research data [19]. Video rating offers many advantages over bedside rating. With video recordings, raters can replay and review observational data as many times as needed. Video rating allows for a level of observation and analysis that may not be attained as a bedside rater. However, video technology may lack the contextual data of real-time, like a bedside observation might provide [19,20,21].

Some technical challenges were identified with the use of video technology. Video recordings may not capture all facial expressions and movements of the patient, and nursing staff in the room often accidentally obstruct the view of the camera. The lighting in the room and the angle of the camera made it difficult for the video raters to recognize tears and face flushing for some patients. Another challenge of video rating was to evaluate muscle tension while viewing the passive flexion and extension of the patient’s arm performed by the bedside rater. This item is easier to assess when performed in real-time at the bedside so that muscle resistance may be felt by the rater. Although videos allowed raters to replay the recordings as many times as needed for observational rating, it may lead to the identification of behaviors that could be missed in real-time observation at the bedside. 

The training appeared adequate to achieve a good interrater agreement between video raters with different backgrounds. Therefore, our results support that this type of training can be provided to people with and without a health-related background, which is consistent with other pain studies in individuals in rehabilitation or with dementia [16,20]. Scoring practice with patient videos and discussing behavioral observations are important aspects of the training. It is worth mentioning that it was more challenging for VR1 (non-health professionals) to recognize ventilator alarms from other sources in the video recordings. Training on the use of CPOT-Neuro is essential for both research and clinical purposes for proper pain assessment in this vulnerable population. The CPOT-Neuro could be further validated in acute or rehabilitation contexts of care. 

### Limitations

The sample was small, limiting the estimation of interrater agreement in the LOC groups, especially the altered LOC and unconscious groups. The sample was also less representative of patients with non-traumatic brain injury. The bedside and video raters were not blinded to procedures and may have observed more behaviors during turning that are known to be painful. ICC could not be obtained for the unconscious group due to the very small sample size. ICCs were good for research purposes but modest for clinical purposes [12].

## 5. Conclusions

The interrater agreement of CPOT-Neuro scores between bedside and video raters was supported for research purposes with ICCs, ranging from 0.65 to 0.80 in this sample of 56 ICU patients with a brain injury. Training strategies should include patient video scoring and a discussion on behavioral observations. The video technology was also useful for examining interrater agreement and could also be used to check intrarater agreement in future studies when interrater agreement is low to locate the source of unreliability (i.e., between and/or within raters) [22].

## Figures and Tables

**Table 1:** Sample description of patient participants.

	Conscious (*n* = 31)	Altered LOC (*n* = 17)	Unconscious (*n* = 8)	Total (*n* = 56)
Male sex: *n* (%)	17 (55%)	13 (77%)	7 (88%)	37 (66%)
Age: median (IQR)	61 (34–79)	60 (28–74)	45.5 (26.5–54.5)	57.5 (33.25–75.5)
Ethnicity: *n* (%)				
Caucasian	24 (77%)	13 (76%)	5 (63%)	42 (75%)
Asian	2 (7%)	2 (12%)	0	4 (7%)
Black	3 (10%)	0	0	3 (5%)
Hispanic	1 (3%)	0	2 (25%)	3 (5%)
Other	1 (3%)	2 (12%)	1 (12%)	4 (7%)
Diagnosis: *n* (%)				
TBI	24 (77%)	13 (77%)	7 (88%)	44 (79%)
Non-TBI	7 (23%)	4 (23%)	1 (12%)	12 (21%)
Mechanical ventilation: *n* (%)	12 (39%)	16 (94%)	7 (88%)	35 (63%)
APACHE: median (IQR)	13 (9–16)	16 (11.5–20.5)	15.5 (10.25–27.5)	14 (9–17.75)

LOC—level of consciousness; IQR—interquartile range; TBI—traumatic brain injury. APACHE—Acute Physiologic Assessment and Chronic Health Evaluation.

**Table 2:** CPOT-Neuro scores (median and IQR) of the bedside and video raters for each procedure.

Procedure	Rater	Conscious (*n* = 31)	Altered LOC (*n* = 17)	Unconscious (*n* = 8)	Total (*n* = 56)
NIBP	Bedside	0 (0–1)	0 (0–0.5)	1 (0–2.75)	0 (0–1.75)
	VR1	0 (0–1)	0 (0–0)	0.5 (0–1)	0 (0–1)
	VR2	0 (0–1)	0 (0–0)	0.5 (0–2.75)	0 (0–1)
Turning	Bedside	3 (1–4)	2 (0–3.5)	3.5 (2–4)	3 (1–4)
	VR1	2 (0–4)	2 (0.5–3)	1.5 (1–2)	2 (1–3)
	VR2	3 (2–4)	3 (1.5–4)	4 (3–4)	3 (2–4)

LOC = level of consciousness; NIBP; non-invasive blood pressure; VR = video rater; bedside (research staff); VR1 (undergraduate student in neuroscience); VR2 (graduate student and experienced research staff).

**Table 3:** Intraclass Correlation Coefficient (95% confidence interval) of CPOT-Neuro scores between pairs of bedside and video raters for each procedure.

Procedure	Pairs of Raters	Conscious (*n* = 31)	Altered LOC (*n* = 17)	Unconscious (*n* = 8)	Total (*n* = 56)
NIBP	Bedside—VR1	0.73 (0.50–0.86)	0.44 (0.04–0.75)	0.62 (0.08–0.91)	0.68 (0.50–0.80)
	Bedside—VR2 *	0.63 (0.26–0.84)	0.65 (0.16–0.89)	---	0.65 (0.40–0.80)
	VR1–VR2	0.69 (0.44–0.84)	0.69 (0.44–0.84)	0.61 (0.09–0.91)	0.69 (0.53–0.81)
Turning	Bedside—VR1	0.79 (0.60–0.89)	0.73 (0.40–0.90)	0.67 (0.02–0.93)	0.74 (0.59–0.84)
	Bedside—VR2 *	0.90 (0.77–0.96)	0.66 (0.16–0.89)	---	0.80 (0.64–0.89)
	VR1–VR2	0.82 (0.67–0.91)	0.78 (0.49–0.91)	0.66 (0.01–0.92)	0.79 (0.66–0.87)

LOC = level of consciousness; NIBP; non-invasive blood pressure; VR = video rater; bedside (research staff); VR1 (undergraduate student in neuroscience); VR2 (graduate student and experienced research staff); * *n* = 35 (including 19 conscious, 13 altered LOC, and 3 unconscious) as VR2 was the bedside rater for 21 patient participants.

## Data Availability

The data presented in this study are available on request from the corresponding author. The data are not publicly available due to ethical guidelines.

## Public Involvement Statement

There was no public involvement in any aspect of this research.

## Guidelines and Standards Statement

This manuscript was drafted following the GRRAS (guidelines for reporting reliability and agreement studies) for reliability and agreement research.
